# Adaptive Response of *Escherichia coli* to Pexiganan: Insights from Genomic Analysis

**DOI:** 10.3390/antibiotics15090825

**Published:** 2026-08-25

**Authors:** Kübra Can Kurt, Liam F. Katzin, Landon Tamaddon, Ali Arslan, Alexander G. Lucaci, Christopher E. Mason

**Affiliations:** 1 Department of Basic Medical Sciences, Hamidiye Faculty of Dentistry, University of Health Sciences, Istanbul 34668, Türkiye; 2 Department of Physiology and Biophysics, Weill Cornell Medicine, New York, NY 10065, USA; 3 Department of Mechanical and Industrial Engineering, Northeastern University, Boston, MA 02115, USA; katzin.l@northeastern.edu; 4 Columbia College, Columbia University, New York, NY 10027, USA; lrt2141@columbia.edu; 5 Department of Molecular Biology and Genetics, Institute of Natural Sciences, Yıldız Technical University, Istanbul 34220, Turkey; aliarslan47@gmail.com; 6 Department of Systems and Computational Biomedicine, Weill Cornell Medicine, New York, NY 10065, USA; agl4001@med.cornell.edu

**Keywords:** antimicrobial peptide, pexiganan, evolutionary adaptation, whole genome sequencing, RNA-seq analysis

## Abstract

**Background/Objectives**: Antimicrobial peptides (AMPs) are considered alternatives to classical antibiotics due to limited resistance development in bacteria. However, bacteria can develop resistance to AMPs through evolutionary adaptation, including oligosaccharide modifications and multidrug efflux pumps. Further research is needed to elucidate the defense mechanisms employed against AMPs to address the emerging resistance problem. Pexiganan is a cationic peptide with effective broad-spectrum antimicrobial activity. The aim of this study is to elucidate the genomic and transcriptomic basis of *E. coli*’s evolutionary adaptation to pexiganan. **Methods**: The *E. coli* ATCC BAA-2523 strain became resistant to pexiganan via evolutionary adaptation methodologies. Whole-genome and transcriptome analyses of resistant and susceptible populations were conducted using Nanopore sequencing and Illumina RNA sequencing, respectively. **Results**: Resistance development became particularly evident after 15 µg/mL, and the bacteria demonstrated the ability to grow even at high doses (up to 1000 µg/mL). Increases in expression levels of classical ARGs such as *emrB, acrF*, *OXA*, *sul2*, and *dfrA14* in the pexiganan-resistant strain indicate that bacteria have the potential for broad-spectrum resistance to other antibiotics alongside pexiganan. Missense mutations have been identified in the phosphatidylserine synthase and cardiolipin synthase genes, which are involved in membrane biosynthesis. Increased expression was observed in the membrane-bound lytic murein transglucosylase (mltF), the murein hydrolase activator (EnvC), and the Antigen 43 (*Ag43*) gene. **Conclusions**: Pexiganan may not only target the cell membrane but also trigger the bacterium’s overall transcriptional response and cross-resistance and MDR systems. Upregulation of multidrug efflux pumps, lytic murein transglucosylase, the murein hydrolase activator and the Antigen 43 gene might be associated with resistance. In addition, the missense mutation was detected in the membrane biosynthesis genes *pssA* and *clsB*. In vivo infection models, targeted functional genomics, and comprehensive phenotypic cross-resistance testing will be required to validate our results.

## 1. Introduction

Currently, public health faces a serious threat due to the rapid spread of pathogens that have developed resistance to traditional antibiotics [1,2,3,4,5]. To overcome this antibiotic crisis, the potential use of antimicrobial peptides (AMPs) as therapeutic agents is being investigated [6,7,8]. AMPs are attracting widespread interest as promising alternatives to classical antibiotics in the fight against infectious diseases [9,10,11]. They offer clinical advantages due to their low immunogenicity, rapid bactericidal effects, and slow development of resistance [12,13,14]. These peptides are expected to be a new generation of therapeutics that are less susceptible to the development of resistance due to their broad-spectrum antimicrobial activity and ability to support the host immune system [15,16,17].

The assumption that pathogens are less likely to develop resistance to antimicrobial peptides (AMPs) than to traditional antibiotics has increasingly been questioned in recent years. Experimental and clinical studies have indicated that bacteria can develop permanent adaptation and resistance to AMPs following prolonged or repeated exposure to them [18,19,20]. Bacteria can develop resistance to AMPs through mechanisms such as modification of oligosaccharides [21], tolerance via biofilm formation or aggregation [22], and the use of multidrug efflux pumps [23]. Thus, the mechanisms of bacterial resistance to AMPs may also trigger cross-resistance to antibiotics [15,20,24,25]. Further research into understanding the defense mechanisms against AMPs is critical for improving their use as therapeutic agents.

Pexiganan is a synthetic AMP derived from magainin [26]. Magainin is a small peptide isolated from the skin of the African clawed frog (*Xenopus laevis*) that exhibits broad-spectrum antimicrobial activity [27], and its bactericidal effect is based on disrupting the structural integrity of the cell membrane, leading to cell death. Pexiganan, also known as MSI-78, is an analogue of magainin-2 and a cationic peptide consisting of 22 amino acids [28,29]. However, details regarding the mechanisms of resistance that may develop against Pexiganan in clinically significant Gram-negative bacteria such as *Escherichia coli* remain limited in the literature. *E. coli* is among the five most deadly pathogens according to the 2019 Global Burden of Disease Study [30]. Newborns and individuals over the age of 60 are particularly severely affected by *E. coli* infections [31,32,33,34]. This pathogen causes a range of infections, including urinary tract infections, pneumonia, peritonitis, meningitis, wound infections, bacteremia, and sepsis [35,36,37,38,39]. Multidrug-resistant (MDR) *E. coli* is a highly lethal pathogen that has developed resistance to most traditional antibiotics, making treatment extremely difficult and sometimes impossible [30,40,41,42]. The aim of this study is to elucidate the genomic and transcriptomic bases of *E. coli*’s evolutionary adaptation to Pexiganan using Nanopore and RNA-seq sequencing.

## 2. Results

### 2.1. Development of Pexiganan Resistance During Experimental Evolution

**Figure 1:**
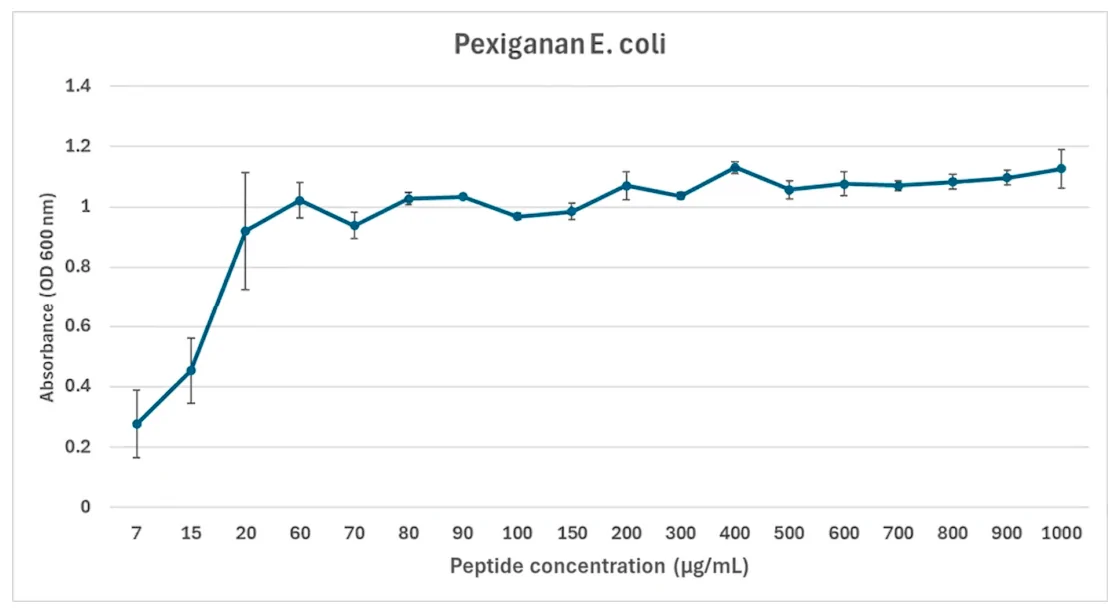
Increasing resistance to Pexiganan in *E. coli* with evolutionary adaptation. Absorbance was measured at 600 nm against increasing peptide concentrations. All experiments were performed in triplicate, and the mean values were presented. The standard deviation is shown on the graph.

Serial passage experiments conducted to evaluate the adaptive resistance of *E. coli* to Pexiganan revealed that bacterial growth changed significantly depending on the peptide concentration (Figure 1). The MIC value specified for *E. coli* is 8 mg/L [43]. Initially, the *E. coli* strain showed growth inhibition at a Pexiganan concentration of approximately 7 µg/mL (Figure 1), which was determined using the MIC test. However, after successive exposure to sublethal concentrations, the bacterial populations were found to develop tolerance to Pexiganan (Figure 1). Throughout the experimental process, the development of resistance became particularly evident at Pexiganan concentrations of 15 µg/mL or above, and bacterial growth was observed to continue with increasing concentrations. OD600 measurements revealed that the bacteria gained an increasingly greater growth capacity in the 15–100 µg/mL range and that this adaptation stabilized over successive generations (Figure 1).

### 2.2. Variation in Minimum Inhibitory Concentration (MIC) Values

Resistant *E. coli* strains, obtained through experimental evolution, and sensitive strains were compared in terms of their susceptibility to Pexiganan using the standard microdilution method. While the MIC value determined for the parental strain was approximately 7 µg/mL, it was found that this value increased to ≥100 µg/mL in the resistant strains obtained through experimental evolution. This increase in MIC values is consistent with the OD600 increase observed during the experimental evolution process, confirming that *E. coli* acquired a high level of phenotypic resistance to Pexiganan. All measurements across three independent biological replicates were consistent (Figure 1).

### 2.3. DNA-Based Identification of Resistance Genes

All genomic data obtained using Nanopore sequencing were analyzed using the DeepARG v2 deep learning algorithm. Bacteria can develop resistance by expelling antimicrobial peptides (AMPs) that have penetrated the cell using efflux pumps [44]. The analyses identified genes associated with multidrug resistance in ancestral and resistant populations, such as mdt and emr operons. Specifically, the genes *emrB, acrF, bpeF*, and *mdtK*, which are associated with active efflux pump systems, were identified in both strains (Table 1).

A single nucleotide alteration causing an Ala74Thr missense mutation was observed in the *pssA* gene, which encodes phosphatidylserine synthase. Furthermore, a single nucleotide change causing a Glu405Lys missense mutation was observed in the *clsB* gene, which encodes cardiolipin synthase B. Both genes play a role in membrane biosynthesis. Single nucleotide changes causing synonymous mutations were observed in the *Rob, psd*, and *clsC* genes; however, as they had no effect on the protein sequence, they were not included in the evaluation. No variants were detected in the canonical charge-modifying genes (*pmrA/pmrB/pmrC, phoP/phoQ, arnBCADTEF, eptA/eptB, mgrB,* and *lpxA/C/D*).

### 2.4. Transcriptomic Analysis of Resistance-Associated Genes

RNA-seq analysis of resistant populations, combined with DeepARG annotation, revealed transcriptional patterns distinct from the genomic profile. While acrF, bpeF, mdtK, and vanH were detected at the RNA level, their expression levels did not significantly increase relative to those of the sensitive strain.

**Figure 2:**
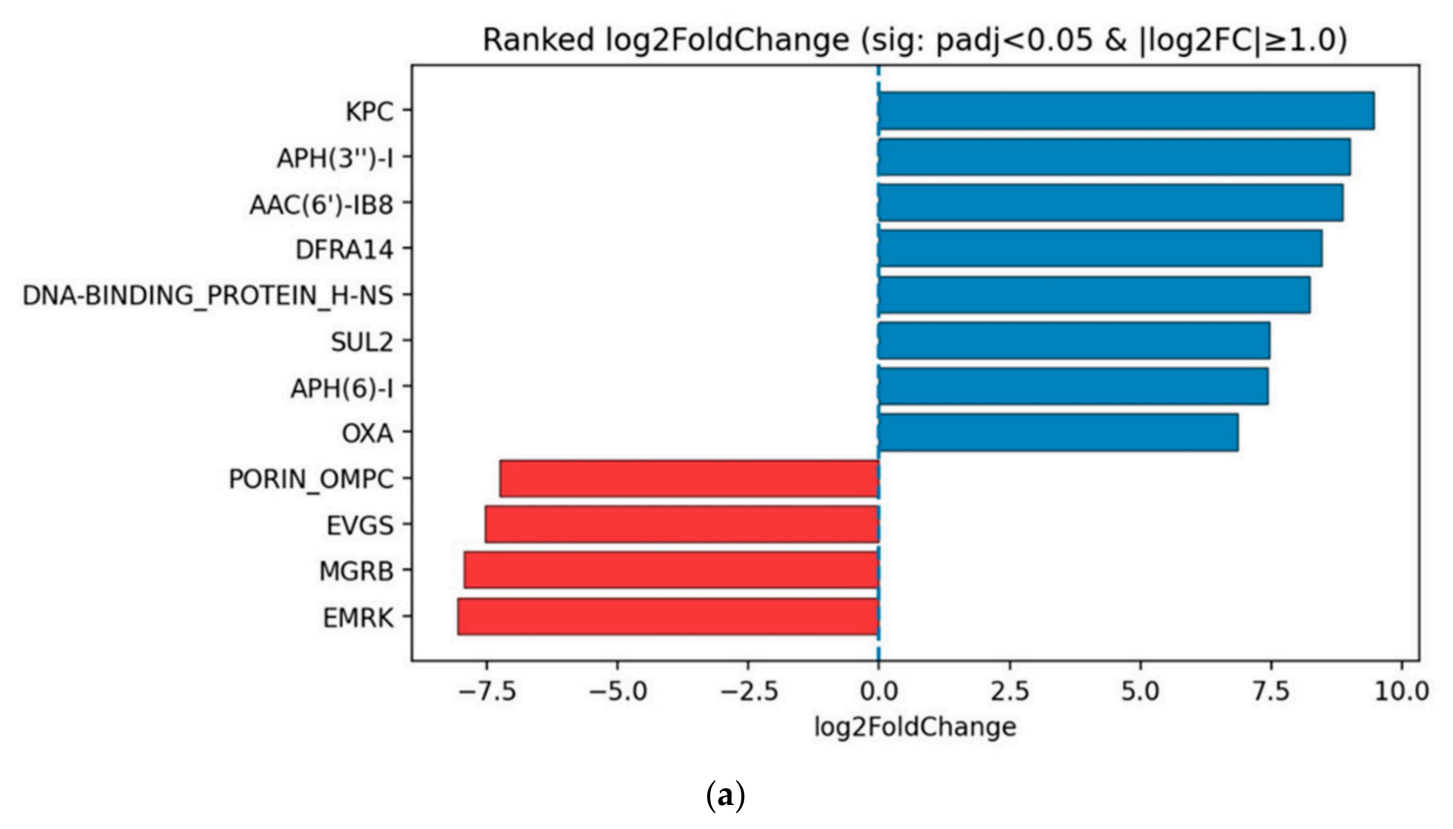

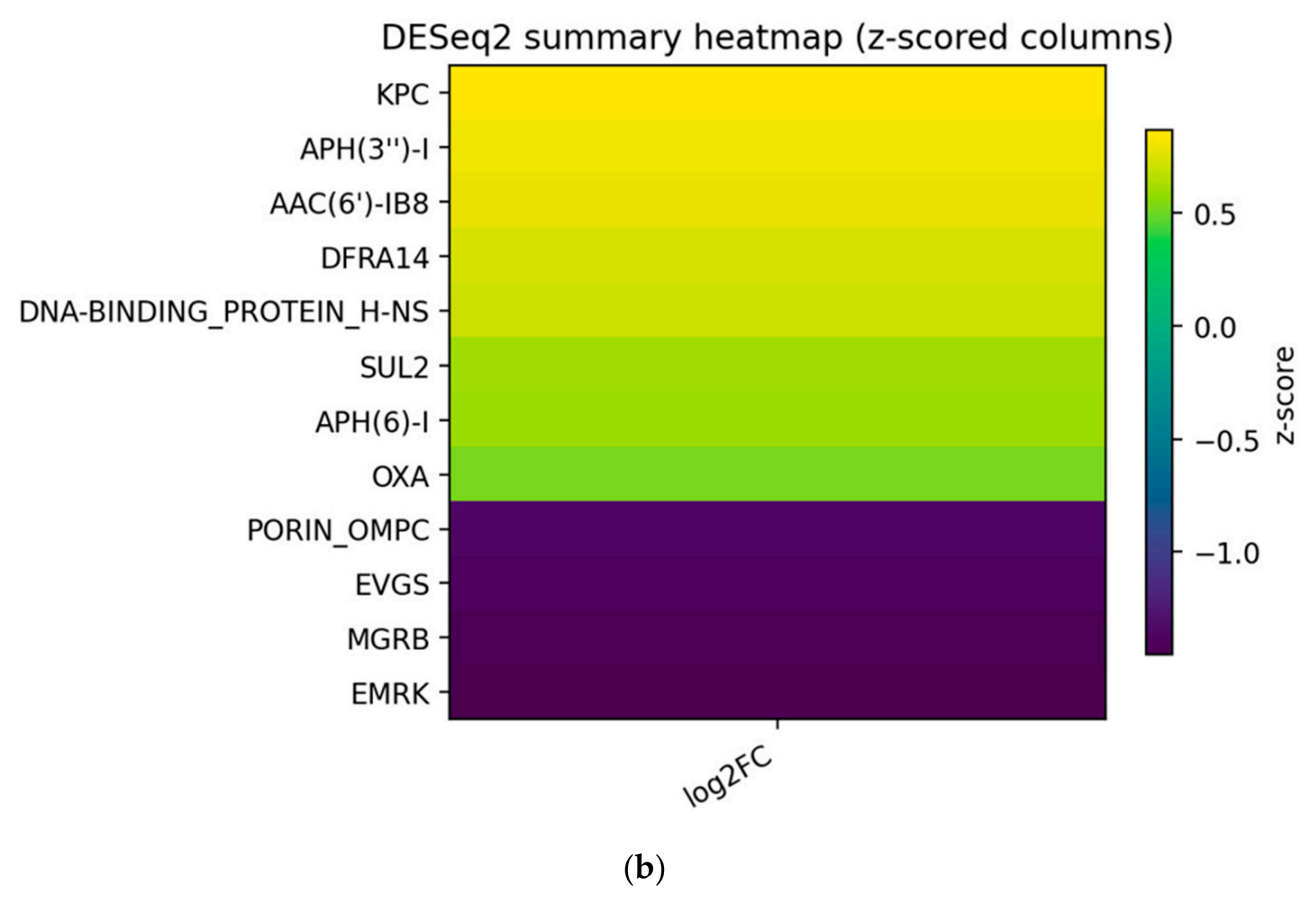
(**a**). Differential gene expression profiling in Pexiganan sensitive and resistant strains. Ranked log2 fold change values of the top 12 significantly deregulated genes. Positive and negative values denote upregulation (blue) and downregulation (red), respectively. Statistical significance was determined using DESeq2 with a threshold of adjusted *p*-value with triplicate samples. (**b**). Heatmap of genes upregulated in response to evolved peptide resistance Data represents relative expression levels (Z-score scaled) of the top differentially expressed genes between Pexiganan sensitive and resistant *E. coli* strain.

In contrast, several resistance-associated genes showed strong transcriptional activation in resistant populations, with 6–10-fold increases in RNA samples (Figure 2a,b). These included KPC and OXA, which are β-lactamases conferring resistance to β-lactam antibiotics; APH(3″)-I, APH(6)-I, and AAC(6′)-IB8, which are aminoglycoside-modifying enzymes; dfrA14 and sul2, which are metabolic resistance determinants; and H-NS, which is a global transcriptional regulator influencing stress responses and multidrug resistance.

It is common for different resistance genes to be co-located in the same region [45]. Furthermore, this co-localization allows for the acquisition of resistance to different agents simultaneously through co-selection. For this reason, ARGs that are not associated with Pexiganan resistance but are highly expressed may have exhibited this behavior because of co-selection. Alternatively, there might be an undiscovered effect on AMPs, such as Pexiganan.

This transcriptional upregulation demonstrates that Pexiganan exposure can selectively activate clinically relevant antibiotic resistance genes, even in the absence of significant changes in efflux pump gene expression (Figure 2).

**Figure 3:**
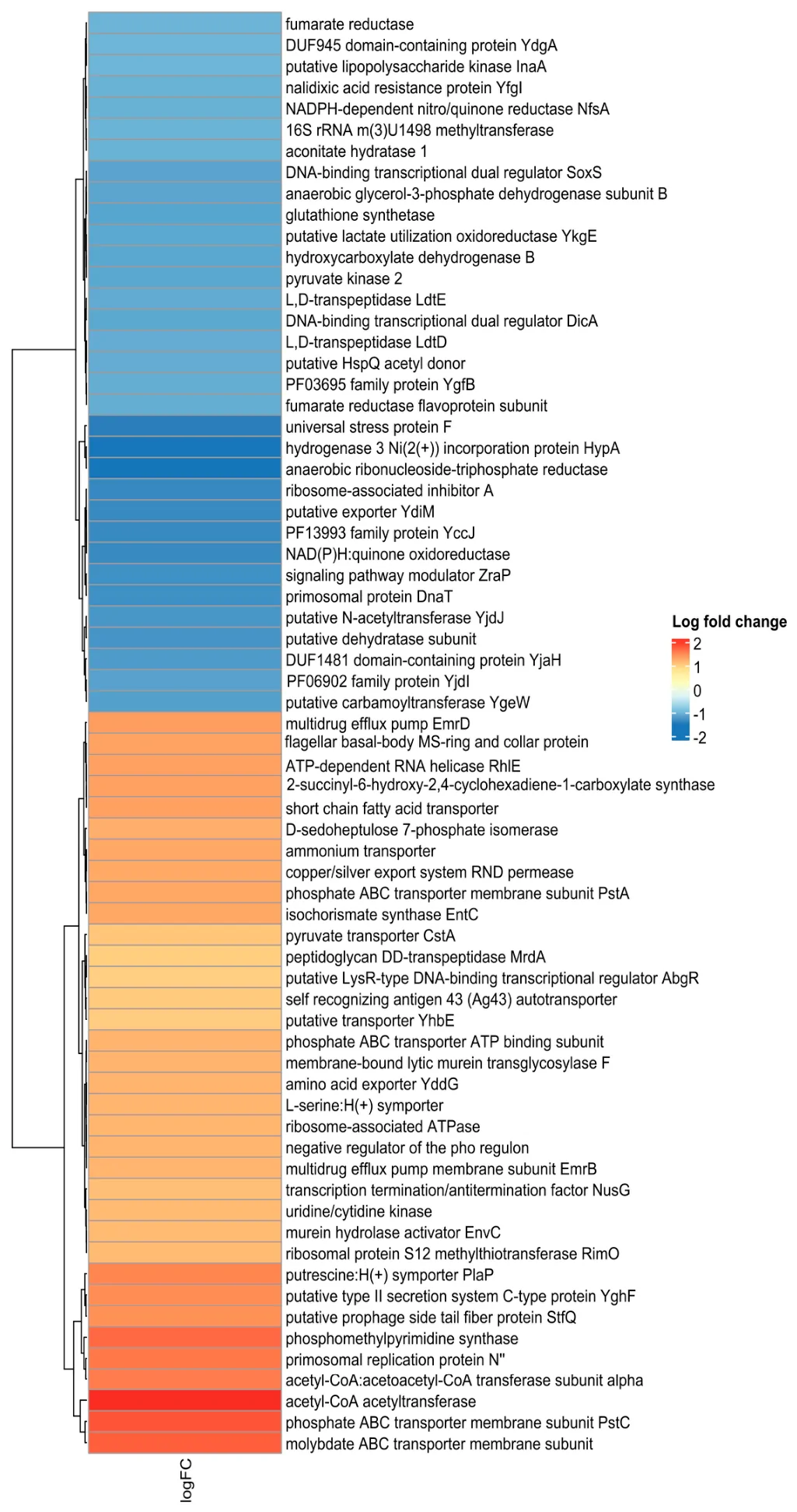
Differential gene expression profiling in Pexiganan sensitive and resistant strains. Ranked log2 fold change values of significantly deregulated genes. Positive and negative values denote upregulation (red) and downregulation (blue), respectively. Statistical significance was determined using DESeq2 with a threshold of adjusted *p*-value with triplicate samples.

In Pexiganan-resistant *E. coli* strains, a more than two-fold increase in expression was observed in the multidrug efflux pump genes *emrD* and *emrB*, the membrane-bound lytic murein transglucosylase (*mltF*) gene, the murein hydrolase activator *envC* gene, and the Antigen 43 (*Ag43*) gene (Figure 3). There was no meaningful difference in the expression levels of the genes responsible for lipid A modification.

## 3. Discussion

Antimicrobial peptides have long been studied as alternatives to classical antibiotics due to their broad spectrum of activity and the assumption that the development of bacterial resistance is relatively difficult [9,46,47]. It has been suggested that AMPs are evolutionarily “resistant” treatment options against antibiotic resistance [48,49,50]. However, recent studies have shown that evolutionary adaptation to AMPs is possible and that resistance can indeed develop [20,51,52,53]. The findings of this study support this view by demonstrating that *E. coli* can develop high levels of resistance to Pexiganan, a cationic AMP, within a short period of time under sublethal and repeated exposure.

In our study, it was demonstrated that the MIC value of the *E. coli* ATCC BAA-2523 populations that developed resistance to Pexiganan through experimental evolution was 14 times that of the parental strain, which was 7 µg/mL. At the end of the experimental evolution process, multiple genes associated with multidrug resistance (MDR), missense mutations in membrane biosynthesis genes, and upregulating efflux pump and membrane bound transglycosylase genes were detected in the *E. coli* ATCC BAA-2523 populations that became resistant to Pexiganan.

Whole-genome DNA analysis using Nanopore revealed the presence of the *emrB, acrF, bpeF, mdtK*, and *vanH* genes in the sensitive and resistant *E. coli* ATCC BAA-2523 populations. These genes are associated with MDR and efflux pump systems, indicating that bacteria have the potential to develop broad-spectrum resistance to other antibiotics in addition to Pexiganan [54,55,56]. Our DNA sequence data suggest that Pexiganan resistance is associated with the activation of the bacterium’s general defense systems rather than a single target-specific alteration. *vanH*, which is normally associated with glycopeptide resistance in Gram-positive bacteria, was detected in this study; it encodes a dehydrogenase involved in the modification of peptidoglycan precursors. These modifications may indirectly hinder the binding of AMPs to the membrane and disrupt membrane integrity by altering the physicochemical properties of the cell wall [20]. Although *vanH* does not directly confer resistance to Pexiganan, it can be considered part of a general stress and resistance response.

A single nucleotide change was observed that causes Ala74Thr and Glu405Lys missense mutations in the *pssA* gene, which plays a role in membrane biosynthesis, and in the *clsB* gene, respectively. Studies conducted over the past two years strongly support the role of membrane phospholipid composition in cationic AMP resistance. In Gram-negative ESKAPE pathogens, it has been shown that the redirection of phospholipid biosynthesis increases tolerance to cationic peptides and colistin [57]. It has been reported that an abundance of cone-shaped, non-bilayer-forming lipids such as cardiolipin and phosphatidylethanolamine directly modulates pore formation by AMPs [58]. Furthermore, it has been shown that the primary target of many newly discovered AMPs is directly anionic phosphatidylglycerol and cardiolipin [59] and that AMP exposure triggers a lipidome reshaping that alters membrane biophysics in bacteria [60]. Our finding in *clsB* and *pssA* could affect Pexiganan resistance, which is consistent with the current literature.

The development of resistance to AMPs is not limited to the modification of membrane lipids; classical resistance mechanisms, such as multidrug resistance (MDR) and efflux pump systems, also contribute [54,55,56]. However, the RNA-seq data showed that the *acrF, bpeF, mdtK*, and *vanH* genes were not transcriptionally active under all conditions. This highlights the difference between genetic potential and functional resistance and indicates that environmental stresses play a critical role in shaping gene expression [61]. The use of efflux pumps as a defense mechanism to pump out cationic peptides could cause Gram-negative bacterial resistance to AMPs [44,62]. However, in the resistant bacteria, a more than two-fold increase was observed in the expression of the *emrD* and *emrB* genes, which encode multidrug efflux pumps. Therefore, the detected *emrD* and *emrB* efflux pump genes may be associated with resistance. The increase in the expression of the putative transporter gene *yhbE* may have indirectly influenced the development of resistance.

Membrane-bound lytic murein transglycosylase (mltF) is responsible for the remodeling of the cell wall in bacteria by cleaving the MurNAc-GlcNAc bond in peptidoglycan. This gene is part of a network associated with stress resistance and membrane homeostasis [63]. A more than two-fold increase in the expression of the membrane-bound lytic murein transglycosylase gene was observed. Given this gene’s role in peptidoglycan degradation and cell wall remodeling, it may have contributed to Pexiganan resistance. Furthermore, the increased expression of the murein hydrolase activator *envC* gene could have activated genes associated with cell wall remodeling in the resistant bacteria.

Another mechanism of AMP resistance is the development of tolerance through biofilm formation and aggregation. Antigen 43 in *E. coli* triggers cell aggregation and biofilm formation and enhances the development of resistance to antimicrobial agents [22]. In Pexiganan-resistant *E. coli* strains, the expression of Antigen 43 increased more than two-fold. The increased expression of the Antigen 43 gene, which is associated with biofilm formation and aggregation, could be linked to Pexiganan resistance.

The *lpxH*, *lpxD*, and *lpxB* genes, which encode UDP-2,3-diacylglucosamine pyrophosphohydrolase, are responsible for lipopolysaccharide A modification in Gram-negative bacteria [64,65]. Increased expression of these genes was not observed in the Pexiganan-resistant *E. coli* strains. The reason for this may be that these genes are no longer required after resistance has developed.

Perron et al.’s [66] study is one of the first to investigate the experimental resistance of *E. coli* and *Pseudomonas fluorescens* bacteria to Pexiganan, and it was observed that mechanisms of hereditary resistance to Pexiganan have evolved. It is also one of the first studies to experimentally disprove the widespread belief that resistance to antimicrobial peptides could not develop. However, it only examined phenotypic resistance (increased MIC values).

Our study not only confirmed the findings of Perron, Zasloff, and Bell [66] but also identified the genetic mechanisms underlying this resistance. The findings obtained in this study are consistent with the framework proposed by Lázár et al. [67], which shows that resistance evolution is often accompanied by increased sensitivity to other conditions. Lázár et al. [67] reported that bacteria that develop antibiotic resistance can exhibit increased sensitivity to different antibiotics and that this comes at an evolutionary cost. Similarly, in this study, although only resistance to Pexiganan was examined, resistant populations were found to experience performance loss in different growth environments and conditions. This suggests that resistance acquisition carries context-dependent fitness costs and that adaptation does not confer an advantage under all conditions. Therefore, the results support Lazar et al.’s view that resistance evolves alongside broad cross-sensitivity and evolutionary constraints [67,68]. Yu, Wang, Zhang, Wang, and Liu [68] evaluated the evolutionary trajectory of bacterial resistance to AMPs using the *E. coli* MG1655 model strain. They found that AMPs showed a lower and slower tendency to develop resistance but that some drug-resistant strains showed increased virulence. Our findings are consistent with their findings [68] of increased virulence but are not consistent with their findings that resistance to AMPs develops very slowly. The development of resistance under continuous sublethal concentration exposure was demonstrated both phenotypically and genotypically.

Genomic (DNA) and transcriptomic (RNA-seq) analyses revealed a clear distinction between the presence of genes associated with antibiotic resistance and their functional activity in Pexiganan-adapted *E. coli* populations. RNA-seq data analysis indicated that exposure to Pexiganan resulted in notable changes in the *E. coli* transcriptome. Compared to the parental strain, the resistant strain showed increased expression of genes associated with various resistance mechanisms. RNA-seq data showed that clinically critical antibiotic resistance genes were activated after Pexiganan exposure. This indicates that the development of resistance depends not only on genetic presence but also on transcriptional regulation under environmental pressures.

Although this study provides comprehensive multi-omics data on the evolution of Pexiganan resistance in *E. coli*, it has some limitations. The study was conducted in vitro under laboratory conditions; this may not fully reflect the complex physiological environments, host–immune interactions, and pharmacokinetic dynamics present during in vivo application. Although the study identified SNPs in genes such as *clsB* and *pssA*, functional genomics studies were not conducted for each variant. Research combining in vivo infection models, targeted functional genomics, and comprehensive phenotypic cross-resistance testing will be valuable for validating and expanding upon these findings.

## 4. Materials and Methods

### 4.1. Experimental Evolution of Pexiganan-Resistant E. coli

In the experiments, we used the *E. coli* ATCC BAA-2523 strain as the “ancestral strain”. The experiment was performed in 96-well plates in a microplate reader at 37 °C. Pexiganan 5 mg (Synonyms: MSI 78 free base, Cat. No.: HY-105088 Purity: 98.66%) was obtained from Medchemexpress (Monmouth Junction, NJ, USA). The well plates were filled with TSB to a total volume of 200 µL. Then, 10 µL of 10^6^ bacteria (excluding the negative control) was added to each well containing 200 µL of medium, resulting in the number of bacteria in each well being 5 × 10^6^ CFU (Colony Forming Units). One of the microplates was used as a negative control. Dilution ratios: Starting from the average MIC (minimum inhibitory concentration) of the peptide in the literature (20%), 10 µL of peptide was added to each well. This plate was incubated at 37 °C for 18–24 h. At the end of the incubation, absorbances were measured at 600 nm on an optical reader. Bacteria that survived and showed growth at the highest peptide concentration were collected and used as inoculum for the next passage. This process was repeated daily to ensure the evolutionary selection of resistant phenotypes. Resistant bacterial strains were collected from the well and stored. The *E. coli* strain was susceptible to Pexiganan in the initial experiments but became resistant over time. The resistant *E. coli* strain was maintained with the addition of glycerol. All experiments were performed in triplicate.

### 4.2. MIC (Minimum Inhibitory Concentration) Test

The MIC value specified for *E. coli* was 8 mg/L [43]. The minimum inhibitory concentration (MIC) for our strain was determined using the micro-dilution method. Briefly, 5 µL (1 × 10^5^ CFU/mL) of mid-logarithmic growth bacterial culture diluted 1:100 was inoculated into 96-well plates containing two-fold serial dilutions of AMPs in 100 µL of TSB. Each experiment was performed in triplicate. The plates were incubated at 37 °C. After 24 h of incubation, the lowest concentration that inhibited visible bacterial growth was defined as the MIC.

### 4.3. DNA Isolation from Resistant Bacterial Strains

Triplicate samples were collected for DNA isolation. DNA was extracted using a Qiagen DNeasy UltraClean DNA isolation kit according to the recommended protocol. The quality and quantity of the DNA extracted were measured using a Nanodrop spectrometer (Thermo Scientific, Waltham, MA, USA) and agarose gel electrophoresis.

### 4.4. RNA Isolation from Resistant Bacterial Strains

Triplicate bacterial samples for each Pexiganan-sensitive and -resistant condition were stabilized using DNA/RNA Shield solution for long-term storage. The total RNA was isolated from bacterial samples using a Quick-RNA Fungal/Bacterial Miniprep Kit (R2014) according to the kit protocol. DNase I treatment was performed to remove residual DNA.

#### RNA-Seq Data Analysis

Three samples were collected from each of the resistant and ancestral strains and subjected to Illumina RNA sequencing using a Zymo-Seq RiboFree Total RNA Library Kit. RNA samples with concentrations ranging from 10 to 250 ng were used for RNA sequencing. cDNA synthesis was performed using random primers. rRNA molecules were removed using the RiboFree universal ribodepletion protocol. After adapter ligation and library amplification, Illumina sequencing was performed at 2 × 150 bp.

Subsequent data analysis was performed using the SAMSA2 v2.2.0 metatranscriptome analysis pipeline [69]. In summary, the resulting paired-end reads were merged using PEAR (v.0.9.8) [70]. Subsequently, adapters and low-quality reads were removed using Trimmomatic (v0.40) [71]. rRNA residues were subsequently removed using SortMeRNA (v4.3.6) [72]. The functional annotation of genes was performed via BLASTx mapping against reference genome proteins using the DIAMOND (v0.8.3) program [73,74] and the *Escherichia coli* K-12 reference strain. The differential expression of genes between the control and resistance-evolved samples was calculated using the DESeq2 package in the R environment [75].

ARG-related gene expression analysis was calculated using the short-read module of the deepARG program. ARGs in the RNA-seq reads were identified using deepARG, and a count table was generated. The differential expression of genes between the control and resistance-evolved samples was calculated using the DESeq2 package in the R environment [75].

### 4.5. Genome Sequencing with Next-Generation Sequencing

To identify the molecular basis of resistance, samples were collected at two distinct stages: the ancestral (sensitive) phase and the evolved (resistant) phase. DNA samples of good quality (260/280 ratio of ~1.8) and in sufficient quantity (1 µg) were sent to be sequenced. Sequencing was performed by Plasmidsaurus (Louisville, KY, USA) using Oxford Nanopore technologies, and bacterial whole-genome sequencing was performed at 100× sequencing depth.

### 4.6. Bioinformatics Analysis

Based on quality scores, the bottom 5% of the worst FASTQ reads were removed via Filtlong v0.2.1 (with default parameters) [76]. The reads were then downsampled to 250 Mb via Filtlong to create a rough sketch of the assembly with Miniasm v0.3 [77]. Using information acquired from the Miniasm assembly, the reads were re-downsampled to ~100× coverage with a heavy weight applied to remove low-quality reads. Then, Flye v2.9.1 assembly [78] with parameters selected for high-quality ONT reads was run, and the Flye assembly was polished via Medaka v1.8.0 (https://github.com/nanoporetech/medaka, accessed on 24 March 2025). Finally, ARGs were detected using the deep learning model DeepARG v2 and assembled genome fasta files [79].

## 5. Conclusions

Exposure to Pexiganan results in high levels of transcriptional activation of the classical ARGs, including KPC, OXA, APH(3″)-I, APH(6)-I, AAC(6′)-IB8, dfrA14, and sul2. H-NS activation is associated with the stress response and global gene regulation; therefore, AMPs such as Pexiganan may not only target the cell membrane but also trigger the bacterium’s overall transcriptional response. In addition, the missense mutation detected in the *pssA* and *clsB* genes, which are responsible for membrane biosynthesis, may have contributed to resistance. Furthermore, the increased expression observed in the multidrug efflux pump genes *emrD* and *emrB*, the membrane-bound lytic murein transglucosylase (*mltF*) gene, the murein hydrolase activator *envC* gene, and the Antigen 43 (*Ag43*) gene may also be associated with resistance. In vivo infection models, targeted functional genomics, and comprehensive phenotypic cross-resistance testing are required to validate our results.

## Figures and Tables

**Table 1:** The efflux pump and cell wall-associated genes detected in Pexiganan-resistant *E. coli* strains and their contributions to multidrug resistance.

Gen	System/TYPE	Function	Contribution to Pexiganan Resistance
*emrB*	MFS efflux	Transport of hydrophobic and amphipathic compounds out of the cell	Prevents intracellular accumulation
*acrF*	RND efflux	Broad-spectrum antibiotic and toxin excretion	Removes AMPs that affect the cell membrane
*bpeF*	RND efflux	Excretes cationic peptides and antibiotics	Provides phenotypic resistance
*mdtK*	MATE efflux	Exports cationic compounds	Reduces intracellular concentrations of AMPs
*vanH*	Cell wall precursor modification	D-Ala-D-Lac synthesis	Reduces the binding of cell wall-targeted AMPs

## Data Availability

Metagenome and RNA-seq raw data are available at the Sequence Read Archive (SRA) under bio projects accession number PRJNA1484553.

## Abbreviations

The following abbreviations are used in this manuscript:

MDR	Multidrug-resistant
AMPs	Antimicrobial peptides
MIC	Minimum Inhibitory Concentration
ATCC	American Type Culture Collection
